# Morphometric Modifications in *Canthon quinquemaculatus* Castelnau 1840 (Coleoptera: Scarabaeinae): Sublethal Effects of Transgenic Maize?

**DOI:** 10.3390/insects8040115

**Published:** 2017-10-21

**Authors:** Victor Michelon Alves, Malva Isabel Medina Hernández

**Affiliations:** Programa de Pós Graduação em Ecologia, Departamento de Ecologia e Zoologia, Universidade Federal de Santa Catarina, Florianópolis 88040-900, Brasil; malva.medina@ufsc.br

**Keywords:** agriculture, body shape, dung beetle, ecology, morphology

## Abstract

The effects of transgenic compounds on non-target organisms remain poorly understood, especially in native insect species. Morphological changes (e.g., changes in body size and shape) may reflect possible responses to environmental stressors, like transgenic toxins. The dung beetle *Canthon quinquemaculatus* (Coleoptera: Scarabaeinae) is a non-target species found in transgenic crops. We evaluated whether *C. quinquemaculatus* individuals inhabiting corn fields cultivated with different seed types (conventional, creole and transgenic) present modifications in body shape compared to individuals inhabiting adjacent native forest fragments. We collected *C*. *quinquemaculatus* specimens across an agricultural landscape in southern Brazil, during the summer of 2015. Six populations were sampled: three maize crop populations each under a different seed type, and three populations of adjacent forests. After sampling, specimens were subjected to morphometric analyses to discover differences in body shape. We chose fifteen landmarks to describe body shape, and morphometric data were tested with Procrustes ANOVA and Discriminant Analysis. We found that body shape did not differ between individuals collected in conventional and creole crops with their respective adjacent forests (*p* > 0.05); however, transgenic crop populations differed significantly from those collected in adjacent forests (*p* < 0.05). Insects in transgenic maize are more oval and have a retraction in the abdominal region, compared with the respective adjacent forest, this result shows the possible effect of transgenic crops on non-target species. This may have implications for the ecosystem service of organic matter removal, carried out by these organisms.

## 1. Introduction

Body shape is directly associated with several important ecological aspects, such as high-speed displacement capabilities, and consequently predator–prey interactions [1,2]. Shape characteristics can give advantages to males in sexual selection, having greater chances of mating [3]. Modifications in body shape offer advantages to certain types of behavior, such as foraging [4,5], reproduction [6] and habitat use [7]. Body morphology is also linked with community structuring since differences in shape are usually associated with different feeding and nesting strategies. Consequently, shape can be associated with better adapted competitors with improved foraging strategies in a particular community [8].

In addition to providing important information on species ecology, body shape may also indicate the effects of possible environmental stressors on individuals. These environmental stressors may be natural, such as ultraviolet radiation [9], or may be anthropic, like pollution [10]. Morphological alterations may imply possible ecological consequences, such as reduction in feed and displacement capacities [11,12]. Thus, in the face of environmental disturbances, body shape alterations may be classified as sublethal effects, and can affect an individual’s fitness [9,13]. Sublethal effects are defined as the physiological or behavioral changes in individuals exposed to chemical compounds [14]. These changes reduce individual fitness and have already been described in various non-target insects such as butterflies [15], bees [16] and dung beetles [17].

Sublethal effects of chemical compounds associated with agricultural practices have been observed in dung beetles. Dung beetles fed with feces containing Ivermectin (a chemical product used to control parasites in cattle) presented behavioral changes, increased developmental time, reduced fecundity and oviposition, and reduced cephalic capsule size [18,19,20]. Behavioral and physiological changes were also described in dung beetles fed with cattle feces containing transgenic maize. When fed with transgenic products, dung beetles showed lower dispersion velocity and alterations in the immune system [21]. These sublethal effects decrease the individual’s fitness, leading to abnormalities in their development, behavior and morphology. Fitness reduction may be evidenced by changes in individual size [22] or shape, particularly in holometabolous insects where the adult stage is greatly influenced by larval development. Therefore, larvae that develop in poor environments, with few resources, or under some kind of specific crop system (e.g., transgenic crops or under insecticidal and fungicidal effect) may result in adults presenting changes in shape and size.

Maize (*Zea mays* L.) is the second largest crop production in the world [23]. Maize production is associated, among other factors, with seed differences. The use of genetically modified seeds is growing globally, reaching approximately 180 million hectares in 2014 [24]. Since the beginning of its implementation in the mid-1980s, questions about their environmental safety have been raised [25]. Some studies have described the effectiveness and apparent safety of transgenic maize in reducing economically important insect populations and not harming non-target organisms [26,27]. On the other hand, several studies have shown target insect resistance development [28], accumulation of transgenic toxin compounds in the food chain [29], and lethal effects on non-target organisms [30]. Conventional seed varieties are severely affected by insects of economic importance, since the combination of food abundance with lack of natural enemies (due to native vegetation suppression) favors overpopulation of phytophagous insects, mainly Lepidoptera larvae. Creole seeds (landraces) normally are less productive than other seeds; however, they have increased genetic variability, and more resistance to phytophagous insects and specific environmental conditions [31]. These landraces can be productive even in unfavorable conditions [32], and represent a genetic heritage since they are selected by the farmers themselves, through the selection of more adapted individuals [33].

*Canthon quinquemaculatus* Castelnau 1840 is a non-target species that feeds on decaying material, and thus is engaged in nutrient cycling, promoting environmental services regulating physico-chemical soil properties [34,35]. *C*. *quinquemaculatus* is a widespread species throughout South America and it feeds on feces and carcasses of mammals and birds. These animals may consume different types of maize. Bt toxin can be transferred through the trophic chain: individuals of *Orius majusculus* (an insect predator) contained Bt toxins when feeding on spider mites reared on transgenic maize [36]. Thus, through the trophic chain these Bt toxins can accumulate and reach *C*. *quinquemaculatus* populations. In addition to the accumulation of transgenic toxins, individuals may suffer from different types of inputs, such as insecticides, which are used in maize crops. These inputs are greater in transgenic and conventional seeds, and are extremely low in creole seeds. Under the assumption that changes in individual shape indicate possible effects on non-target species physiology, the aim of this study was to evaluate whether *C*. *quinquemaculatus* individuals present morphometric differences in populations found within different maize crops (conventional, creole, and transgenic seeds) when compared with populations found in adjacent control populations inhabiting forest remnants.

## 2. Materials and Methods

**Figure A1:**
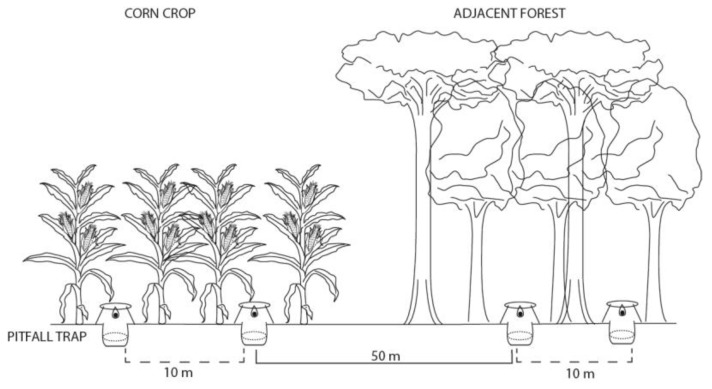
Experimental design scheme used for the captured individuals of *C. quinquemaculatus* in maize crops and adjacent forests of Atlantic Forest in the region of São Miguel do Oeste, SC, Brazil.

Population sampling occurred in the region of São Miguel do Oeste, Santa Catarina State, Brazil (26°43′31″ S, 53°31′05″ W). The region is composed of a mosaic of Atlantic forest fragments and maize crops. Local climate is humid subtropical (Cfa) according to the Köppen classification, with an annual average temperature between 16.3 °C and 17.9 °C, and rainfall between 1790 mm and 2280 mm [37]. Samples were collected during the summer of 2015 in maize crops with different profiles: five areas of conventional maize, five areas of creole maize, and five areas of transgenic maize. Furthermore, 15 adjacent forest remnants, one for each crop area, were sampled in order to have an independently replicated control population for each type of crop. The insects were collected using ten pitfall traps per area, five baited with human feces (10 g) and five with pig carrion (10 g) during 48 h. The traps were spaced 10 m apart within the same area, and a minimum distance of 50 m was established between crop traps and forest fragments to avoid interference (Appendix A Figure A1). Captured insects were fixed in 70% alcohol and deposited in the entomological collection of the Federal University of Santa Catarina (UFSC).

From a total of 231 specimens of *C*. *quinquemaculatus* collected, we used 157 individuals for morphometric analyses. We tried to balance the proportion between males and females in the most equitable way possible. Thus, we measured 12 specimens from conventional maize crops (6♀, 6♂); 17 from creole crops (11♀, 6♂); 28 from transgenic crops (14♀, 14♂); 36 from forest fragments adjacent to conventional crops (18♀, 18♂); 30 from forest fragments adjacent to creole crops (10♀, 20♂); and 34 from forests adjacent to transgenic crops (17♀, 17♂).

**Figure 1:**
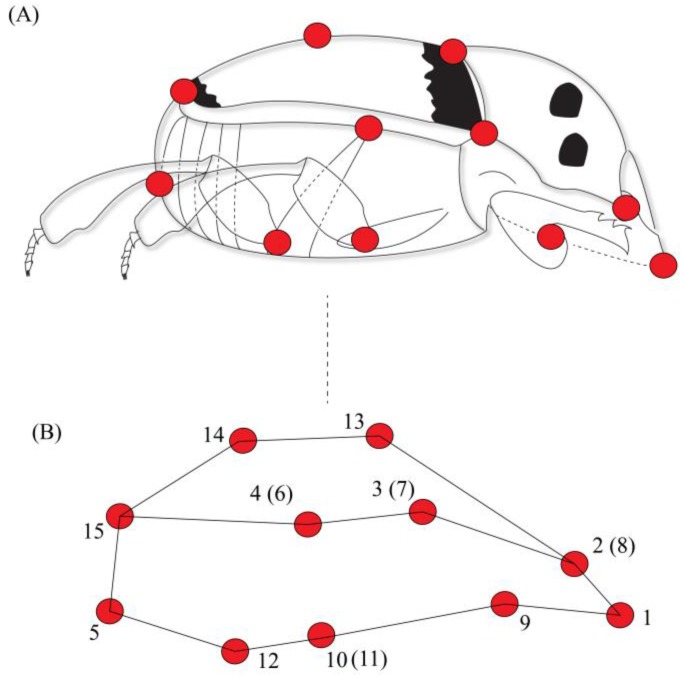
Landmarks (red dots) used in shape analysis of *C*. *quinquemaculatus*. (**A**) Lateral view; (**B**) Graphical representation of body shape based on 15 landmarks, adapted from Hernández et al. [8]. Landmarks in parentheses correspond to the same region on the other side of the body.

We defined the body shape through the placement of 15 anatomical landmarks. These 15 landmarks were chosen because they capture all morphological variations describing body shape in both anteroposterior and ventral dorsal axis, and these landmarks have already been successfully used to describe dung beetle body shape [8]. Each landmark corresponded to a point in space defined by three-dimensional cartesian coordinates (x, y, z) [38] as follows: (1) anterior margin of the head; (2) eye position; (3) division between the pronotum and the elytra; (4) division between the thorax and the abdomen; (5) posterior margin of the abdomen; (9) point of insertion of the anterior legs; (10 and 11) points of insertion of the central legs; (12) point of insertion of the posterior legs; (13) anterior point of convergence between elytra; (14) central point (mid-line) of convergence between elytra; and 15) posterior margin (along the mid-line) of the elytra. Points 6, 7 and 8 corresponded to points 4, 3 and 2 for the other side of the body, respectively [8]. Insects were photographed using Canon T3i camera in dorsal, ventral and lateral view; therefore, body shape was captured in three dimensions (Figure 1). A standard protocol for photographing specimens was established: insects were photographed on millimeter paper on a fixed surface to minimize the position effects. The camera was attached to a tripod and kept at a distance of 10 cm from each specimen at a perpendicular angle. Each individual had a tag, and for digitalization of the landmarks the individuals were mixed, so the marking was blind.

Coordinates from each landmark underwent Generalized Procrustes Analysis (GPA). The GPA is a three-step multivariate technique that assesses body shape eliminating the effects of size, position and orientation. The first step of the GPA is to center each landmark at the cartesian origin, eliminating the position effect. Afterwards, the landmark coordinates are scaled, eliminating the effect of size. Lastly, the landmark coordinates are rotated around the origin, which removes the orientation effect. Through these three steps, size and orientation data are removed leaving only body shape information [39]. We performed a canonical variable analysis (CVA) to view each individual’s position in the multivariate space. The overall shape difference was primarily tested with a Procrustes ANOVA, and to test the differences between maize crop populations and adjacent forest populations we performed a discriminant analysis (DA) based on the Mahalanobis distance. The reliability of a population’s individuals was determined by cross-validation to calculate the correct percentage of classification of the individuals in the original population, based only on the morphometric variation. Anatomical landmarks were inserted into specimens with the softwares TpsDig 2.16 [40] and tpsUtil1.53 [41]. The GPA, CVA and DA were performed in Morpho J 1.05d software [42].

## 3. Results

**Figure 2:**
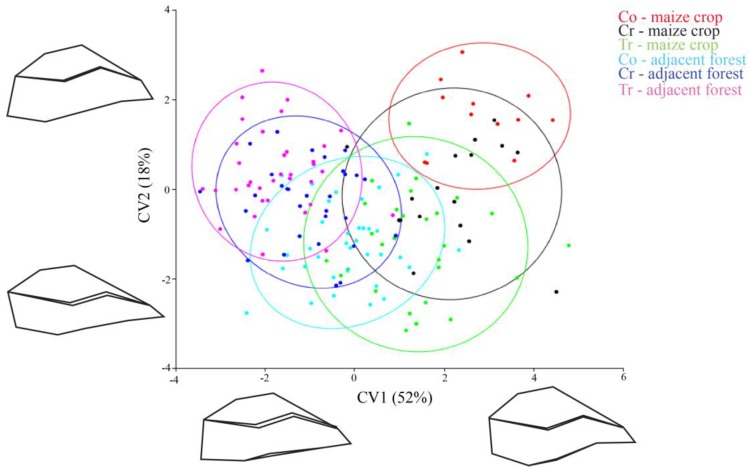
Position of *C*. *quinquemaculatus* populations in morphometric space formed by two main axes of Canonical Variable Analysis, collected in the region of São Miguel do Oeste, SC, Brazil. Conventional (Co), Creole (Cr) and Transgenic (Tr); ellipses indicate a 95% confidence interval.

The shape of the individuals was assessed from the 15 landmarks and each specimen was represented by a point in Figure 2. Together, the first (CV1) and second (CV2) canonical variables in the CVA explained 70% of the observed body shape variation. The CVA showed that maize crop populations have mostly positive values in CV1 (red, black and green points in the Figure 2). On the other hand, forest fragment populations were mostly negative (light blue, blue and pink) and inferred a shape distinction between the insects that inhabit maize crops and those from the adjacent forests.

The CV1 (which accounted for 52% of the variance) organized individuals in a flat (negative scores) to oval (positive scores) body shape gradient; forest insects corresponded to a flat body shape and maize crop insects to an oval body shape (see the population’s shape design in both corners of Figure 2). Populations with positive CV1 (maize crop populations) (body shape in the right corner of Figure 2) had the landmarks of the dorsal region displaced above average shape, and landmark 5 (corresponding to abdominal region) displaced towards the body center. Together, these landmark displacements indicated a pronounced oval shape and an abdominal contraction (Figure 2 right corner). Populations with negative scores in CV1 (forest fragment populations) had landmark 5 (abdominal region) dislocated away from body center, and had the landmarks of the dorsal region displaced toward the body center. These rearrangements resulted in a flattened body shape (Figure 2, left corner).

Regarding CV2, there was no clear distinction between insects, insects with positive scores had dorsal region landmarks displaced towards the body center, and the ventral region landmarks dislocated away from the body center. These landmark displacements indicated a retraction on the anterior–posterior axis of body (Figure 2, designs at the top of CV2). Insects with negative scores had the dorsal region landmarks displaced away from the body center, and the ventral region landmarks dislocated towards the body center. This landmark configuration indicated an extension of the body’s anterior–posterior axis (Figure 2, designs at bottom of CV2).

The cross-validation tests indicated that an average of 75% (ranging between 58% and 90%) of individuals were correctly allocated to their respective populations. Maize crop populations had a high validation (ranging between 64% and 90%), including transgenic maize crop populations with 82% (Table 1).

**Figure 3:**
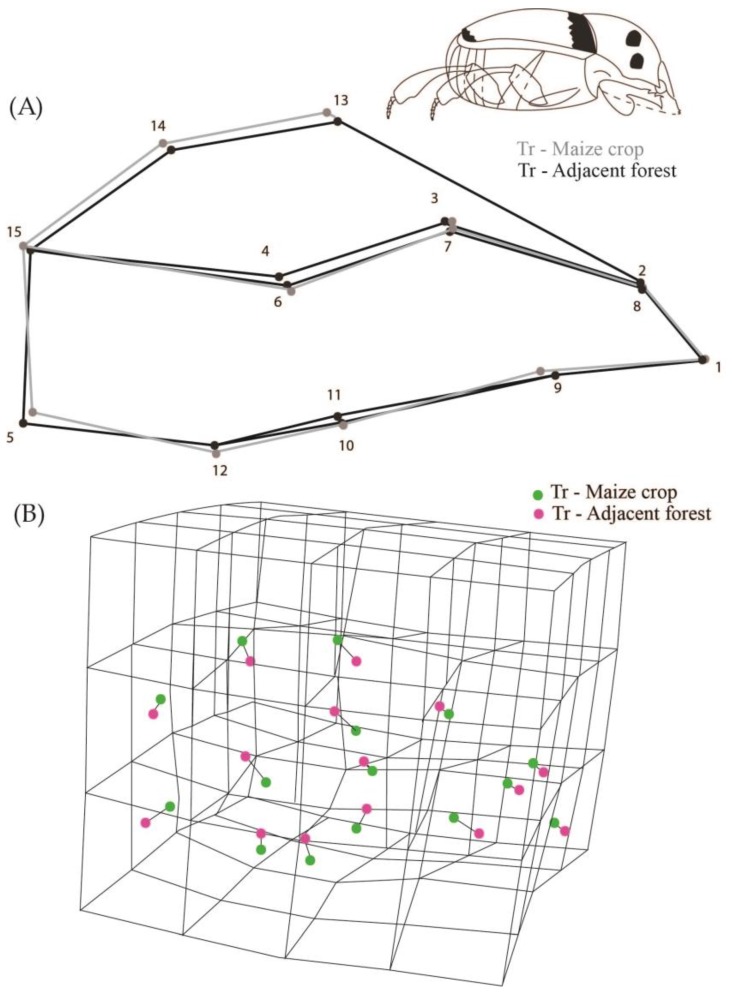
(**A**) 2D Graphical reconstruction of *C*. *quinquemaculatus* body shape. Gray lines show body shape of transgenic maize crop populations, and black lines show adjacent forest populations; (**B**) 3D Graphical reconstruction of *C*. *quinquemaculatus* body shape. Green dots: transgenic maize populations. Pink dots: adjacent forest populations.

While analyzing all the populations we observed that they were different in relation to body shape (Procrustes ANOVA, F = 2.28, Pillai’s Trace = 2.03, *p* < 0.0001). We observed that the shape of the populations of creole and conventional crops were not significantly different from their respective adjacent forest areas (DA, *p* > 0.05). However, populations from transgenic maize crops differed in body shape from the populations of adjacent forests (DA, *p* = 0.0002) (Figure 3). The transgenic maize crop populations have the dorsal region landmarks higher up than the average shape of insects from adjacent forests (landmarks 13, 14 and 15 in Figure 3), and landmark 5 shifted to the center of the body. Consequently, this configuration indicates that populations of transgenic crops had a more oval shape and an abdominal retraction compared to individuals from adjacent forest populations (Figure 3).

## 4. Discussion

We observed different body shape patterns for *C*. *quinquemaculatus* individuals living in different habitats; populations found in transgenic maize crops presented significant morphometric modifications when compared to adjacent forest fragments. To date no published studies have reported changes in the entire body shape in response to the use of transgenic organisms in an agricultural environment. Studies with *Diabrotica virgifera virgifera* C. (Coleoptera: Chrysomelidae) collected in different types of maize crop, including transgenic crops, showed that the head of males inhabiting in transgenic maize was significantly smaller than in other treatments [43]. Similarly, individuals of *Papilio machaon* L. (Lepidoptera: Papilionidae) fed with transgenic pollen had lower body mass [22]. These results indicated that immature stages (larvae) could be exposed to sublethal doses of toxic transgenic maize, resulting in smaller adults. While implications of body size as a result of environmental effects are well documented [44], ecological implications of changes in body shape remain poorly known.

Although we did not detect changes in the body size of *C*. *quinquemaculatus*, we found differences in adult morphometrics, leading us to consider that the larvae or adults may have suffered effects derived from feeding on transgenic toxins; furthermore, laboratory experiments are needed to test for differences in the morphology of adults fed with transgenic toxins. With respect to body size, several studies failed to detect changes in insect populations grown in transgenic crops [45,46]. Inability of body size and mass to detect transgenic effects on non-target insect species suggests that both variables are less sensitive than shape, and using body shape to detect sublethal effects may be more efficient and informative. Changes in shape might have a major genetic influence compared to changes in size and mass, which are mainly driven by environmental factors [47].

Changes in body shape or in the shape of functional traits (e.g., wings, legs) can greatly affect some ecological attributes like sexual selection [48], adaptation [49], competition [8], development [50], and predation [51], causing significant changes in important populational parameters such as density, morphological variation, fitness, evolution and survival. Very few studies have evaluated changes in body shape or in the shape of bodily structures in response to transgenic compounds. Rahman et al. [52] observed alterations to skull shape in transgenic tilapia fish (*Oreochromis niloticus*). Studies with Pacific Salmon (*Oncorhynchus* spp.) showed changes in the shape of the head, tail and abdominal regions in response to a transgenic insertion [53]. Studies with transgenic Coho Salmon (*O*. *kisutch*) showed body shape differences between transgenic individuals and wild individuals even if they developed at the same temperatures [12]. This shape difference observed in *Oncorhynchus* spp. can affect its ability to swim, escape from predators and migrate. Similarly, Dunham et al. [54] found body shape differences in transgenic common carp (*Cyprinus carpio*). Furthermore, animals fed with transgenic organisms showed morphometric alterations; rats fed with transgenic potatoes presented shape alterations in the ileum [55]. Shape alterations in genetically modified organisms have also been described for plants; for example, Armon et al. [56] found differences in the leaf shape of wild and genetically modified *Arabidopsis* sp. specimens, suggesting that gene manipulation may interfere with growth regulating systems.

We have indications that transgenic compounds accumulating in the trophic chain affect the dung beetles, causing abnormalities in their physiology and behavior [21]. This pattern may be a product of a sum of factors, like historical management or use of different kinds of insecticides. Habes et al. [57] demonstrated morphometric alterations in *Blatella germanic* ovaries after insecticide application. Moreover, the management associated with transgenic crops alters the structure of the dung beetle community, reducing species abundance [30]. Therefore, the results from *C*. *quinquemaculatus* have given us another clue as to how transgenic compounds may be affecting dung beetles. As observed for *C*. *quinquemaculatus*, changes in insect morphometrics may lead to a decrease in insect fitness in response to the use of transgenics.

## 5. Conclusions

We have shown that the transgenic maize populations of *C*. *quinquemaculatus* are associated with changes in body shape, which may be a possible sub-lethal effect of the transgenic crop in this non-target species. These effects may have repercussions on the ecosystem functions performed by these insects, such as organic matter removal and regulation of soil physico-chemical properties.

## Figures and Tables

**Table 1:** Percentage of correct classification of *C*. *quinquemaculatus* populations based on body morphology and discriminant analysis (DA).

Population	Allocation Value (%)
Conventional—Maize crop	90.66
Creole—Maize crop	64.70
Transgenic—Maize crop	82.14
Conventional—Adjacent forest	58.33
Creole—Adjacent forest	73.33
Transgenic—Adjacent forest	82.85
Overall classification accuracy	75.33
